# Sociodemographic Correlates of Meeting US Department of Health and Human Services Muscle Strengthening Recommendations in Middle-Aged and Older Adults

**DOI:** 10.5888/pcd11.140007

**Published:** 2014-09-18

**Authors:** Jesse W. Vezina, Cheryl A. Der Ananian, Edward Greenberg, Jonathan Kurka

**Affiliations:** Author Affiiliations: Jonathan Kurka, Cheryl A. Der Ananian, Edward Greenberg, Arizona State University, Phoenix, Arizona.

## Abstract

**Introduction:**

A growing body of evidence demonstrates the health benefits of muscular strength training. Physical activity recommendations encourage all adults to participate regularly in muscle strengthening activities. The purpose of this study was to examine the prevalence of meeting the US Department of Health and Human Services (DHHS) muscular strengthening recommendations by middle-aged and older adults and the sociodemographic characteristics associated with meeting these recommendations, using data from the 2011 Behavioral Risk Factor Surveillance System (BRFSS).

**Methods:**

Data from the 2011 BRFSS were used to examine the prevalence of meeting the DHHS muscle strengthening recommendations by adults older than 45. Simple and multiple regression analyses were used to examine the sociodemographic characteristics associated with meeting the recommendations.

**Results:**

Of respondents to the muscle strengthening question (N = 333,507), 79,029 (23.7%) reported meeting the muscle strengthening recommendations. Respondents who were female (odds ratio [OR] = 0.80; 95% confidence interval [CI] 0.78–0.83), widowed (OR = 0.69; 95% CI, 0.66–0.72), 85 or older (OR = 0.63; 95% CI, 0.58–0.68), Hispanic (OR = 0.73; 95% CI, 0.67–0.78), with a body mass index of 30.0 kg/m^2^ or higher (OR = 0.47; 95% CI, 0.45–0.49), and with less than a high school education (OR = 0.32, 95% CI, 0.30–0.35) were less likely to meet the recommendations than their counterparts.

**Conclusion:**

Sociodemographic characteristics such as sex, age, education, and race/ethnicity are significantly associated with meeting the muscle strengthening recommendations, suggesting a need to create tailored interventions and messages to promote participation in strength training.

## Introduction

Recommendations for physical activity from both the American College of Sports Medicine/American Heart Association (1) and the US Department of Health and Human Services (DHHS) (2) emphasize regular participation in muscle strengthening activities for adults. These organizations recommend that adults perform muscle strengthening activities targeting all major muscle groups at least 2 days per week. Enhancing participation in muscle strengthening exercise is critical for meeting the physical activity recommendations, because adults are less likely to meet strength training recommendations than aerobic exercise recommendations. A 2011 study indicated that 29.3% of US adults met strength training recommendations, whereas 51.6% met the recommendations for aerobic activity (3). Understanding factors influencing strength training participation is critical for enhancing participation and tailoring interventions.

The health benefits of muscular strength training are relevant to the aging population. Muscular strength training increases muscle mass (4,5) and improves the physical function (6,7) of adults throughout the lifespan and may decrease the likelihood of developing sarcopenia (the loss of skeletal muscle mass and function). Muscular strength training has positive effects on bone health (8,9), blood pressure (10,11), and insulin sensitivity (12,13). Furthermore, increased muscular strength is inversely correlated with both all-cause and cancer mortality (14). Despite these benefits, only 21.7% of US adults older than 65 meet the muscle strengthening guidelines (3). Data indicate that the US population is living longer (15), and promoting strength training to middle-aged and older adults may have a significant effect on health outcomes.

The primary objectives of this study were to describe the prevalence of meeting the DHHS muscle strengthening recommendations among middle-aged and older adults (adults aged 45 years or older) using self-reported data from the 2011 Behavioral Risk Factor Surveillance System (BRFSS) and to examine the associations between sociodemographic characteristics (age, sex, education, income, race/ethnicity, body mass index [BMI], health status, and marital status) and meeting the recommendations.

## Methods

### Data

Data from the 2011 BRFSS (N = 504,408) were analyzed to examine prevalence estimates of meeting the DHHS muscle strengthening recommendations by middle-aged and older adults (2). The BRFSS is a state-based, annual telephone survey that uses random-digit dialing to collect health data from noninstitutionalized adults older than 18 years. Data were collected by all 50 states, the District of Columbia, Puerto Rico, the US Virgin Islands, and Guam. In the 2011 survey, both landline and cellular telephones were called, but only 1 adult per household was surveyed. Cellular phones were included in the survey to better represent the underrepresented demographic populations (such as those who do not have landline telephones) and to better match known demographic characteristics of the population. The survey contains questions pertaining to sociodemographic characteristics and health behaviors associated with chronic disease, infectious disease, and injury. To assess participation in muscle strengthening activities, respondents were asked, “During the past month, how many times per week or per month did you do physical activities or exercises to strengthen your muscles? Do not count aerobic activities like walking, running, or bicycling. Count activities using your own body weight like yoga, sit-ups or push-ups, and those using weight machines, free weights, or elastic bands.” Possible answers included the number of times per week, per month, or never. It was noted if respondents indicated they didn’t know the answer or refused to answer the question. 

Frequency of activity per month was converted into frequency per week by dividing monthly frequency by the number of weeks in a month. Data on respondents who did not answer or answered “don’t know” to the muscle strengthening question (n = 28,697) or who were younger than 45 (n = 128,534) were excluded from the analysis. Consistent with the recommendations of both the American College of Sports Medicine/American Heart Association (1) and DHHS (2), participants (n = 333,507) were classified as meeting the muscle strengthening recommendations if they reported having participated in muscle strengthening activities 2 or more times per week.

Additionally, the survey assessed whether individuals met the aerobic training guidelines. These data were acquired by asking a series of questions: “During the past month, other than your regular job, did you participate in any physical activities or exercises such as running, calisthenics, golf, gardening, or walking for exercise?” “What type of physical activity or exercise did you spend the most time doing during the past month?” “How many times per week or per month did you take part in this activity during the past month?” and “When you took part in this activity, for how many minutes or hours did you usually keep at it?” Participants were allowed to choose from more than 70 listed activities or describe another activity they participated in. They were also asked this series of questions twice, to assess more than one form of physical activity. The total amount of time spent in physical activities was calculated to determine whether respondents met the physical activity recommendations.

### Statistical analysis

Per BRFSS instructions, before analysis all data were weighted using a technique known as raking (16). Raking allows new variables to be included in the weighting process (telephone source, education level, marital status, and renter/owner status) in addition to the variables that are usually used (race/ethnicity, region within states, age group by sex, sex by race/ethnicity, and age group by race/ethnicity) (16). Data were analyzed by demographic characteristics and weighted to provide prevalence estimates (Table 1); 95% confidence intervals (CIs) were calculated for each estimate.

**Table 1:** Demographics of Participants Older Than 45 Who Answered the Muscle Strengthening Question (N = 333,507), Behavioral Risk Factor Surveillance System Survey, 2011

Characteristic	Unweighted No. (Weighted %)^a^	Met Strength Training Recommendations^b^	
		Unweighted No.	Weighted % (95% CI)
**Sex**			
Male	128,041 (46.9)	33,495	25.9 (25.5–26.4)
Female	205,466 (53.1)	45,534	21.9 (21.6–22.3)
**Marital status**			
Married/member of unmarried couple	184,998 (62.6)	46,769	24.9 (24.5–25.2)
Divorced/separated	60,856 (16.5)	14,256	23.4 (22.7–24.1)
Widowed	61,007 (12.6)	11,496	18.6 (18.0–19.1)
Never married	25,597 (8.3)	6,189	24.6 (23.4–25.7)
**Age, y**			
45–54	85,016 (36.9)	22,288	26.0 (25.4–26.5)
55–64	104,043 (29.7)	25,490	23.7 (23.7–24.2)
65–74	79,568 (18.3)	18,486	23.0 (22.5–23.6)
75–84	49,840 (12.0)	10,034	20.2 (19.5–20.8)
≥85	15,040 (3.1)	2,695	18.1 (17.0–19.3)
**Education**			
Less than high school diploma	30,131 (15.0)	3,866	14.4 (13.5–15.2)
High school graduate or equivalent	102,240 (30.4)	17,417	18.5 (18.0–19.0)
Some college	88,290 (28.6)	20,540	25.0 (24.5–25.6)
College graduate	112,200 (26.0)	37,033	34.2 (33.7–34.7)
**Race/ethnicity**			
Non-Hispanic white	272,013 (74.5)	65,622	24.2 (23.9–24.5)
Non-Hispanic black	24,176 (9.5)	5,280	24.1 (23.0–25.2)
Hispanic	18,501 (10.4)	3,307	18.8 (17.7–19.9)
Multiracial/other	15,340 (5.7)	3,935	27.0 (25.4–28.7)
**Income, $**			
<15,000	36,643 (12.2)	5,818	16.0 (15.2–16.8)
15,000–24,999	53,283 (17.8)	9,488	17.8 (17.1–18.5)
25,000–34,999	35,591 (11.7)	7,254	19.8 (18.9–20.6)
35,000–49,999	44,070 (14.4)	10,097	22.6 (21.8–23.3)
≥50,000	118,219 (43.9)	36,641	30.6 (30.1–31.1)
**Body mass index (kg/m^2^)**			
Underweight (<18.5)	5,002 (1.3)	1,243	24.2 (21.8–26.5)
Normal weight (18.5–24.9)	102,805 (30.0)	30,600	30.2 (29.6–30.8)
Overweight (25.0–29.9)	120,464 (38.4)	29,328	24.9 (24.4–25.3)
Obese (≥30.0)	92,287 (30.3)	15,650	16.9 (16.4–17.4)
**Self-perceived health**			
Good or better	257,002 (76.3)	67,443	26.5 (26.2–26.8)
Fair or poor	75,220 (23.7)	11,313	15.2 (14.7–15.7)

Abbreviation: CI, confidence interval.

a Values may not sum to total because of missing data.

b Individuals were determined to have met the strength training requirements if they self-reported participation in muscle strengthening activities at least twice per week.

Associations between meeting the muscle-strengthening recommendations and the sociodemographic variables of sex, marital status (married or member of unmarried couple, divorced or separated, widowed, never married), age (45–54, 55–64, 65–74, 75–84 and ≥85), education (less than high school diploma, high school graduate, some college, college graduate), race (non-Hispanic white, non-Hispanic black, Hispanic, multiracial or other), income (<$15,000; $15,000–$24,999; $25,000–$34,999; $35,000–$49,999; ≥$50,000), BMI (in kg/m^2^)(underweight (<18.5), normal weight (20.0–24.9), overweight (25.0–29.9), obese (≥30), and self-perceived health (good or better, fair or poor) were examined with simple and multiple regression analyses. The income variable was omitted from multiple regression analysis due to collinearity with education. Participants whose data were missing or who answered “don’t know” to any of the demographic variables were excluded from the respective analyses. In all, data on 315,097 respondents were analyzed in the multiple regression model. All data were analyzed using SAS 9.2 (SAS Institute Inc). All statistical analyses were 2-sided, and differences were considered significant at *P* < .05.

## Results

Of the 333,507 participants who provided strength training data, 53.1% were female, 33.4% were aged 65 years or older, 26.0% were college graduates, 74.5% were non-Hispanic white, 43.9% reported earning $50,000 or more annually, 68.7% were classified as overweight or obese (BMI ≥25.0), and 76.3% reported good or better health (Table 1). Overall, 24% percent of adults aged 45 or older reported meeting the DHHS muscle strengthening recommendations. 

The odds of meeting the recommendations for muscle strengthening activities varied considerably by sociodemographic characteristic (Table 2). Simple and multiple logistic regression analyses indicated that the odds of meeting the strength training recommendations were 22% lower (odds ratio [OR] = 0.78; 95% CI, 0.76–0.81; *P* < .001) for women than men, declined with increasing age (*P* < .001 across all age groups) and as education levels decreased (*P* < .001 across all education levels). Compared with respondents aged 45 to 54 years, the odds of meeting the strength training recommendations were 10% lower for respondents aged 55 to 64 years (OR = 0.90; 95% CI, 0.86–0.93; *P* < .001) and 8% lower for respondents aged 65 to 74 years (OR = 0.92; 95% CI, 0.88–0.96; *P* < .001). The odds of meeting the strength training recommendations were nearly 20% lower (OR = 0.81; 95% CI, 0.77–0.86; *P* < .001) among respondents aged 75 to 84 years and 31% lower among respondents aged 85 or older (OR = 0.69; 95% CI, 0.63–0.75; *P* < .001) than among respondents aged 45 to 54 years. Similarly, the odds of meeting the strength training recommendations were 60% lower among respondents who did not graduate from high school (OR = 0.40; 95% CI, 0.37–0.44; *P* < .001) than among college graduates.

**Table 2:** Odds Ratios of Strength Training Participation, by Sociodemographic Variables, Behavioral Risk Factor Surveillance System, 2011

Characteristic	Simple Logistic Regression		Multiple Logistic Regression	
	OR (95% CI)	*P* Value	OR (95% CI)	*P* Value
**Sex**				
Male	1 [Reference]			
Female	0.80 (0.78–0.83)	<.001	0.78 (0.76–0.81)	<.001
**Marital status**				
Married/member of unmarried couple	1 [Reference]			
Divorced/separated	0.92 (0.89–0.97)	<.001	1.05 (1.00–1.10)	.05
Widowed	0.69 (0.66–0.72)	<.001	0.94 (0.90–0.99)	.02
Never married	0.98 (0.92–1.05)	.62	1.02 (0.95–1.09)	.67
**Age, y**				
45–54	1 [Reference]			
55–64	0.88 (0.85–0.92)	<.001	0.90 (0.86–0.93)	<.001
65–74	0.85 (0.82–0.89)	<.001	0.92 (0.88–0.96)	<.001
75–84	0.72 (0.69–0.76)	<.001	0.81 (0.77–0.86)	<.001
≥85	0.63 (0.58–0.68)	<.001	0.69 (0.63–0.75)	<.001
**Education**				
Less than high school diploma	0.32 (0.30–0.35)	<.001	0.40 (0.37–0.44)	<.001
High school graduate or equivalent	0.44 (0.42–0.45)	<.001	0.49 (0.47–0.51)	<.001
Some college	0.64 (0.62–0.67)	<.001	0.70 (0.68–0.73)	<.001
College graduate	1 [Reference]			
**Race/ethnicity**				
Non-Hispanic white	1 [Reference]			
Non-Hispanic black	0.99 (0.94–1.06)	.84	1.22 (1.15–1.31)	<.001
Hispanic	0.73 (0.67–0.78)	<.001	0.99 (0.92–1.07)	.83
Multiracial/other	1.16 (1.06–1.27)	<.001	1.01 (0.92–1.11)	.85
**Income, $**				
<15,000	0.43 (0.40–0.46)	<.001	—	
15,000–24,999	0.49 (0.47–0.52)	<.001		
25,000–34,999	0.56 (0.53–0.59)	<.001		
35,000–49,999	0.66 (0.63–0.70)	<.001		
≥50,000	1 [Reference]			
**Body mass index (kg/m^2^)**				
Underweight (<18.5)	0.74 (0.65–0.84)	<.001	0.91 (0.80–1.04)	.17
Normal weight (18.5–24.9)	1 [Reference]			
Overweight (25.0–29.9)	0.76 (0.74–0.79)	<.001	0.75 (0.72–0.78)	<.001
Obese (≥30.0)	0.47 (0.45–0.49)	<.001	0.50 (0.48–0.52)	<.001
**Self-perceived health**				
Good or better	1 [Reference]			
Fair or poor	0.50 (0.48–0.52)	<.001	0.66 (0.63–0.69)	<.001

Abbreviations: OR, odds ratio; CI, confidence interval; — , not included.

Simple regression models indicated that the odds of meeting the muscle strengthening recommendations were lower among divorced and widowed respondents than those who were married or a member of an unmarried couple (*P* < .001). However, in multiple regression models the odds of meeting the strength training recommendations were 6% lower among widowed respondents (*P* = .02), while the odds were slightly increased among divorced respondents (OR = 1.05; 95% CI, 1.00–1.10; *P* = .05) compared with respondents who were married or part of an unmarried couple. 

Multiple logistic regression analyses suggested that the odds of meeting the strength training recommendations were higher for non-Hispanic blacks (OR = 1.22; 95% CI, 1.15–1.31; *P* < .001) than for non-Hispanic whites. In both simple and multiple regression analyses, overweight and obese respondents had reduced odds of meeting the strength training recommendations compared with normal weight respondents (all *P* < .001). Finally, in both simple and multiple regression analyses, respondents who perceived themselves as having fair or poor health had significantly lower odds of meeting the recommendations (both *P* < .001).

Further analysis of respondents who answered both the strength training question and the physical activity question (n = 315,097) indicated that the odds of meeting the strength training recommendations were 2.73 times higher among those who met the aerobic physical activity recommendations (OR = 2.73; 95% CI, 2.63–2.83; *P* < .001) compared with those who did not meet the aerobic recommendations. Only 17.2% of adults in this sample met both the physical activity and strength training recommendations. Of those meeting the strength training recommendations, 73.0% met both guidelines. The inclusion of meeting the aerobic physical activity guidelines (yes/no) in the multiple regression model for strength training did not change the relationship of the other sociodemographic variables regarding likelihood of meeting the strength training recommendations (results not shown).

## Discussion

Strength training by middle-aged and older adults is critical for promoting health, functional fitness, and functional independence (17). Overall, our findings suggest that regular participation in muscle strengthening exercises is low (24%) in middle-aged and older adults. Moreover, there was a significant decline in the prevalence of strength training from middle age to the oldest old, with only 18% of respondents aged 85 or older reporting regular participation in strength training. Our findings are consistent with those of other studies that show a decline in strength training with age (3,18,19).

Some evidence suggests that participation in strength training by older adults is increasing. During the past 15 years, older adult participation in muscle strengthening activities has risen by more than 13% (3,19); however, these studies collapsed all older adults into an age category of 65 years or older. Little is known about trends across age categories among older adults. Given the anticipated growth in the number of people living beyond the age of 85, examining whether strength training participation varies by age in older adults (eg, youngest old vs oldest old) is important. As people age, the need for increased participation in strength training is critical for optimal physical function and health outcomes (17). Participation in strength training can reduce illness (14) and promote independent living among older adults (17), which may be especially important for people in the oldest age category, as functional decline occurs at a higher rate among older adults (20).

Participation in strength training by middle-aged and older adults varied by sociodemographic and health characteristics, suggesting the need to tailor communication and prevention strategies to enhance participation. Consistent with findings of other studies (21,22), respondents of low socioeconomic status (low income or education level) were less likely to engage in strength training than their counterparts, and men were more likely than women to engage in strength training. Additionally, respondents who were overweight or obese and those who perceived their health was poor or fair were less likely to meet the guidelines for strength training. Given the high prevalence of overweight and obesity among older adults (19,23), the high prevalence of chronic illnesses among older adults (17), and the potential health benefits of strength training, it is evident that identifying the barriers and enablers of strength training and strategies to enhance participation is essential for improving health outcomes of older adults.

In our study, non-Hispanic blacks were more likely than non-Hispanic whites to meet the strength training recommendations but only in multiple logistic regression analyses. Likewise, Hispanics were less likely than whites to meet strength training recommendations in simple logistic regression analyses, but no differences were observed in multiple logistic regression analyses. These findings suggest that variables other than race/ethnicity such as income and education level, health status, or obesity may have a stronger influence on strength training participation. The National Center for Educational Statistics reported a discrepancy in educational attainment between minorities and whites (24). Nearly 92% of non-Hispanic whites attain at least a high school education compared with 85% of non-Hispanic blacks and 64% of Hispanics. The differences are more pronounced when looking at the percentage of people who obtain a college degree. Roughly 34% of non-Hispanic whites graduate from college whereas only 20% of non-Hispanic blacks and 14% of Hispanics attain this educational level (24). Education is a powerful sociodemographic predictor of health outcomes (25) and is consistently associated with engagement in physical activity (26,27). Education could be an important influence strictly due to knowledge about or skill in strength training or it could be a marker of access and availability. Similarly, ethnic minority populations including Hispanics and Latinos have higher levels of overweight and obesity, which may confound the relationship between race/ethnicity and strength training participation.

Muscle strengthening activities are essential in the maintenance of health and physical function (17). This fact is of particular importance in the aging population, given the high association of aging with decreases in skeletal muscle size and function (28) and the decline in participation in strength training activities. Continuing to develop interventions to target adults throughout their lifespan, including well beyond the age of 65, may offer a strategy to attenuate the effects of sarcopenia and increase physical function in advancing age (29) while also helping to control other cardiometabolic risk factors. Given what is known about the health-related benefits of physical activity (1) and about muscle strengthening activities (30), it is important to increase understanding of the correlates and determinates of strength training participation in older adults. This knowledge could help researchers and practitioners develop tailored approaches or strategies to prevent or attenuate the risk of developing sarcopenia in older adults through enhanced participation in strength training.

Our study has limitations. First, the cross-sectional design of the study did not allow causality to be inferred among any of the variables. Second, the data we used were self-reported and are subject to reporting bias. Also, the BRFSS question that referred to muscle strengthening activities was vague. The question leaves room for interpretation as to what constitutes muscle strengthening activities and does not get at the other part of the recommendations, which specifies the necessary volume (sets/repetitions) that should be performed for maintenance of health.

Our findings indicate that there continues to be a precipitous decline in participation in muscle strengthening activities associated with aging, and that this decline does not stop at age 65. Furthermore, many other sociodemographic variables such as income, education, race/ethnicity, weight, and health status appear to be predictive of participation in muscle strengthening activities. A strong relationship exists between the decline in skeletal muscle strength and physical function of older adults (17), and our findings can be used to develop public service messages aimed at decreasing age-related health risks. The information gained through this research could be used to help identify which sociodemographic subgroups are most in need of such interventions.
